# Development of an Open-source and Lightweight Sensor Recording Software System for Conducting Biomedical Research: Technical Report

**DOI:** 10.2196/43092

**Published:** 2023-02-17

**Authors:** Michael Single, Lena C Bruhin, Narayan Schütz, Aileen C Naef, Heinz Hegi, Pascal Reuse, Kaspar A Schindler, Paul Krack, Roland Wiest, Andrew Chan, Tobias Nef, Stephan M Gerber

**Affiliations:** 1 Gerontechnology and Rehabilitation Group ARTORG Center for Biomedical Engineering Research University of Bern Bern Switzerland; 2 DomoHealth SA Lausanne Switzerland; 3 Department of Sport Science University of Bern Bern Switzerland; 4 Department of Neurology Inselspital, Bern University Hospital University of Bern Bern Switzerland; 5 Support Center for Advanced Neuroimaging (SCAN) University Institute of Diagnostic and Interventional Neuroradiology Inselspital, Bern University Hospital, University of Bern Bern Switzerland

**Keywords:** sensor recording software, on-demand deployment, digital measures, sensor platform, biomedical research

## Abstract

**Background:**

Digital sensing devices have become an increasingly important component of modern biomedical research, as they help provide objective insights into individuals’ everyday behavior in terms of changes in motor and nonmotor symptoms. However, there are significant barriers to the adoption of sensor-enhanced biomedical solutions in terms of both technical expertise and associated costs. The currently available solutions neither allow easy integration of custom sensing devices nor offer a practicable methodology in cases of limited resources. This has become particularly relevant, given the need for real-time sensor data that could help lower health care costs by reducing the frequency of clinical assessments performed by specialists and improve access to health assessments (eg, for people living in remote areas or older adults living at home).

**Objective:**

The objective of this paper is to detail the end-to-end development of a novel sensor recording software system that supports the integration of heterogeneous sensor technologies, runs as an on-demand service on consumer-grade hardware to build sensor systems, and can be easily used to reliably record longitudinal sensor measurements in research settings.

**Methods:**

The proposed software system is based on a server-client architecture, consisting of multiple self-contained microservices that communicated with each other (eg, the web server transfers data to a database instance) and were implemented as Docker containers. The design of the software is based on state-of-the-art open-source technologies (eg, Node.js or MongoDB), which fulfill nonfunctional requirements and reduce associated costs. A series of programs to facilitate the use of the software were documented. To demonstrate performance, the software was tested in 3 studies (2 gait studies and 1 behavioral study assessing activities of daily living) that ran between 2 and 225 days, with a total of 114 participants. We used descriptive statistics to evaluate longitudinal measurements for reliability, error rates, throughput rates, latency, and usability (with the System Usability Scale [SUS] and the Post-Study System Usability Questionnaire [PSSUQ]).

**Results:**

Three qualitative features (event annotation program, sample delay analysis program, and monitoring dashboard) were elaborated and realized as integrated programs. Our quantitative findings demonstrate that the system operates reliably on consumer-grade hardware, even across multiple months (>420 days), providing high throughput (2000 requests per second) with a low latency and error rate (<0.002%). In addition, the results of the usability tests indicate that the system is effective, efficient, and satisfactory to use (mean usability ratings for the SUS and PSSUQ were 89.5 and 1.62, respectively).

**Conclusions:**

Overall, this sensor recording software could be leveraged to test sensor devices, as well as to develop and validate algorithms that are able to extract digital measures (eg, gait parameters or actigraphy). The proposed software could help significantly reduce barriers related to sensor-enhanced biomedical research and allow researchers to focus on the research questions at hand rather than on developing recording technologies.

## Introduction

Digital sensing devices have become increasingly popular in modern biomedical research. They allow researchers to gain objective insights into people’s behavior, helping them gain a deeper understanding of health and disease processes [1]. Sensor-based assessment is particularly relevant in the context of chronic conditions, such as neurodegenerative diseases, which are inherently difficult to assess and have previously often relied on biased, subjective data, such as questionnaires [2]. Despite the significant potential of these new digital technologies, adopting sensor-enhanced biomedical solutions is not simple. Specifically, significant barriers related to technical expertise as well as associated costs exist. As a result, their use is still largely limited to specialized research groups. One possible reason for this is that existing technologies and frameworks that could be used to record internet of things–driven real-world sensor data often require extensive technical expertise, and are frequently in the form of complex and rather expensive solutions (eg, cloud [3] or on-premises deployments). Such solutions are useful on a large scale but may deter many research groups that do not have access to the necessary expertise or resources. There is therefore a strong need for simple yet reliable software to build systems that are operational on cost-efficient consumer-grade hardware as basic as a Raspberry Pi 4 (Raspberry Pi Foundation), and can integrate multiple sensing devices, all while conforming to the clinical and technical requirements of biomedical research settings.

Digital sensing devices are becoming increasingly important, especially in the field of biomedical research, as their assessed measures provide objective and continuous insights into people’s everyday lives [4-7]. This is supported by a multitude of studies that have demonstrated how health-related digital measures can contribute to a deeper understanding of a person’s health and well-being [8]. For instance, digital measures have been used to monitor the progression of neuropsychiatric disorders [9-11], gait abnormalities (eg, speed, stride length, and gait symmetry) [12-14], and cardiovascular indices such as heart rate variability in older adults with dementia [15]. A key advantage of sensor-derived digital measures is the ability to obtain continuous information, as more traditional on-site clinical visits tend to provide only an often biased snapshot of a person’s health status [16]. This access to timely health-relevant information could also allow for the earlier detection of health-related deterioration, which is often associated with decreased health care costs, better health outcomes, and a higher quality of life [17,18]. Beyond research, the availability of real-time sensor data could help lower health care costs by reducing the frequency of clinical assessments conducted by specialists and improve access to health assessments (eg, for people living in remote areas or older adults living at home).

Although the use of digital sensing devices has proven straightforward in laboratory settings [19], their use in the real world is associated with economic, technical, and regulatory issues, which are nontrivial and pose significant obstacles [20]. These circumstances are especially relevant for researchers conducting early stage research (eg, feasibility studies) or with limited resources. Buying a complete sensor system with all devices and applications from a single vendor is not always a viable option either technically or economically. An alternative strategy, developing a dedicated data acquisition system for every research question that relies on sensing devices, can also be impractical in the context of research. In the realm of technical challenges, one major aspect is related to big data requirements, such as volume, velocity, and variety [21]. Early stage research might not inherently contribute to big data, but it may eventually produce high-frequency data streams that generate large volumes of data that must be ingested in (near) real-time. Typically, such high-frequency sensor systems produce several hundred samples per second [22]. These sensor streams can come from numerous sensor types, leading to a variety of data formats. Thus, ensuring high availability and fault tolerance is critical to guarantee stable and high-quality data recordings. Another consideration is that data are sometimes not processed synchronously or are lost due to latency in the network. This problem is exacerbated by the lack of standardization in communication protocols, which makes it difficult to ensure compatibility between sensing devices and related applications across different vendors. Traditionally, cloud-first solutions, thus solutions that were designed with cloud deployments in mind, have been used to tackle the aforementioned challenges in the context of biomedical research using digital sensing devices. Prominent systems that take this approach are SPHERE [23], CART [24], and Waggle [25]. However, such cloud-first solutions, while certainly powerful for large-scale deployments, can quickly become cumbersome and expensive, which often translates into significant maintenance and configuration overhead. Lastly, on-premises deployments may be preferred, especially in the context of medical data, such as recordings in hospitals. A comprehensive listing of such on-premises solutions can be found in the systematic review of mobile and wearable sensing frameworks by Kumar et al [26]. This review provides a detailed comparison of various functional (eg, data storage method) and nonfunctional (eg, extensibility, scalability) features of mobile health (mHealth) sensor systems. Although listed systems such as RADAR-base [27] and AWARE [28] support the integration of external sensor systems, are open source, and ensure scalability (eg, by relying on technologies such as the Apache Kafka platform), they cannot easily be deployed on consumer-grade hardware due to their complexity. However, alternative options are either too specific (ie, they can only be used for certain applications or support only certain sensors) [29] or too general [30], making them challenging to adapt to various uses. There is therefore a significant need for on-premises solutions that address the technical challenges, are extensible, and can be run on consumer-grade hardware.

According to best practice principles on the internet of things [31,32], the ideal sensor system software supports a simple mechanism for integrating multiple and diverse high-frequency data streams from various sensing devices with stability guarantees and minimal development effort. To achieve such a technical solution, the gap between laboratory systems and consumer-grade installations must be bridged. This may best be achieved by an open, scalable, and flexible software infrastructure, as well as a set of tools that enables the integration of data from a wide range of technologies. To this end, we have developed a software system that offers a simple and flexible installation and supports arbitrary sensor technologies. Therefore, the objective of this work is to report on the end-to-end development, functionality, and performance evaluation of this open-source sensor recording software (SRS).

## Methods

### Overview

**Figure 1:**
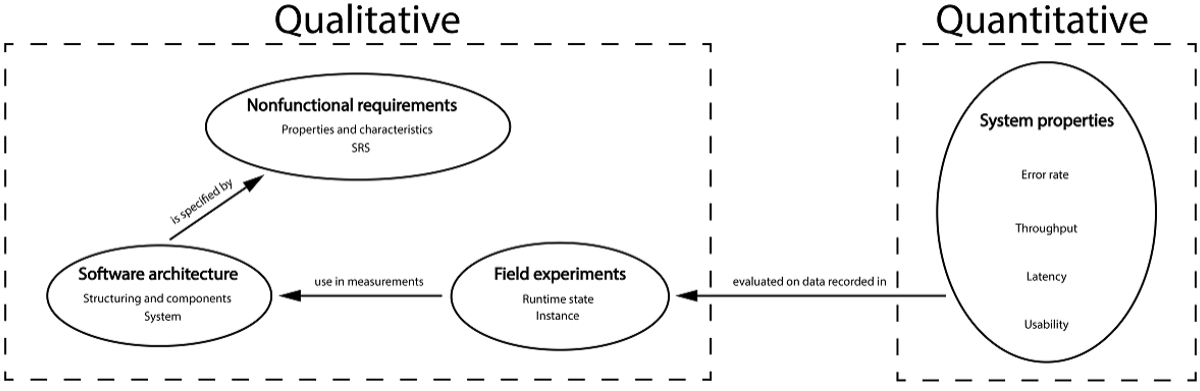
Outline of the qualitative and quantitative aspects of the methods section. First, a conceptualization of the system’s characteristics and properties in the form of nonfunctional requirements is provided, followed by a report on architectural components of its software and their interactions. This is followed by an explanation of the data acquisition in the field experiments; these data were used to evaluate the performance of the system’s properties by applying various metrics (error rate, throughput, latency, and usability). SRS: sensor recording software.

This section details the end-to-end development of the proposed SRS and how its performance was evaluated (Figure 1). The first part describes qualitative aspects of the software system, whereas the second part describes quantitative aspects in terms of system properties. The “Nonfunctional Requirements” section specifies the properties and characteristics of the developed system’s software architecture. The “Software Architecture” section describes the system’s components, how they are structured, and how they interact with each other. The “Field Experiments” section describes the acquisition of data to evaluate the SRS system, which involves running versions of the proposed software, referred to as *instances*. In the “System Properties” section, a documentation of the metrics to evaluate the performance of the system properties is provided.

### Nonfunctional Requirements

The technical aspects of a software system (ie, its technical properties and constraints) can be described in terms of nonfunctional requirements. In the context of software engineering, nonfunctional requirements are defined as the constraints imposed on a system that specify the quality attributes of the software [33]. These requirements therefore affect how the software architecture of a system is designed and implemented. To design the software architecture of the SRS, the nonfunctional requirements and attributes in Textbox 1 were considered.

**Nonfunctional requirements and attributes.**

**1. Reliability**

- Allow the sensor recording software (SRS) to operate without failure while maintaining a specified level of performance for a prolonged period. The software should be fault tolerant, should have a low failure rate, and should be recoverable.

**2. Data integrity**

- Assures integrity, consistency, and correctness of the sensor data in the SRS.

**3. Scalability**

- Handle an increase in workload by adding resources to the system (eg, persisting measurements of high-frequency data producers).

**4. Performance**

- The time it takes for the SRS to accomplish a task (eg, the delay), or the number of requests the SRS completes within a given amount of time (eg, throughput).

**5. Flexibility**

- The degree to which the SRS can be adapted to different configurations without having to change its software, such as the integration of new sensor devices.

**6. Usability**

- Produce a straightforward software system for starting sensor measurements that works on a wide range of hardware solutions and requires only a minimum of technical know-how.

**7. Security**

- Prevent unauthorized access to measured sensor data.

**8. Privacy**

- Controls how sensitive data of the measured individuals are viewed and used.

### Software Architecture

#### Software Architecture: Design

The design of the SRS architecture was derived from the specified nonfunctional requirements by attempting to fulfill several related objectives (Table 1), thus ensuring these objectives directly affect the implementation of the software architecture.

To perform the actual implementation of the SRS architecture, the main technologies and frameworks listed in Textbox 2 were chosen.

**Table 1:** Listing of nonfunctional requirements, their objectives, the sensor recording software attempts to fulfill them, and detailed information about the strategy on how to meet the corresponding objective in the software architecture’s implementation.

Nonfunctional requirement and its objectives			Implementation strategy in software architecture
**Reliability**			
	Prolonged runtime using fault-tolerant policies	Perform system-wide exception handling and apply reactive restart strategies for critical program components in case of system crashes [34]	
**Data integrity**			
	High data quality	Validate all measured sensor samples before and after persisting with the data [35]	
	Reproducible experiments	Have replicable deployment configuration [36]	
**Scalability**			
	System performs as expected under increasing or heavy workloads	Structure the software as a decentralized system in the form of lightweight microservices [37] Rely on technologies that have proven to be scalable [38]	
**Performance**			
	High data throughput rates for handling a large quantity of data streams	Use a concurrent web server capable of handling multiple connections [39] Persist with time-series data in an optimized database technology that supports fast writing operations [40]	
**Flexibility**			
	Easy integration of new sensor devices or third-party applications	Incorporate new sensor-data formats by adhering to the extendable data interface [41] System components are divided into functional parts with high cohesion and low coupling [42]	
**Usability**			
	Supports a large variety of hardware systems	Rely on standard lightweight virtualization technique for the orchestration of microservices [43]	
	Streamline the installation and configuration of the software	Follow best practices for deployment strategies, specifically through version control systems in combination with continuous integration provided by repository hosting services [44]	
	Offers an intuitive method to control sensor measurements	Implement a graphical user interface designed to carry out measurement and the monitoring of the data stream [45]	
**Security**			
	Data protection concerns	Database is encrypted and hosted locally [46] Data can only be accessed through communication channels that use network certificates [47]	
**Privacy**			
	Protect privacy of measured individuals	No identifying information is stored in the database [48]

**The main technologies and frameworks for the actual implementation of the sensor recording software architecture.**

**1. The software-virtualization platform *Docker***

- This virtualization platform Docker [49] offers a standardized, lightweight virtualization technique controlling the execution states of a program (eg, starting, stopping, or restarting a program) in the form of containers leveraging the orchestration of microservices [43]. This aspect substantially simplifies the deployment process, while the level of technical expertise required is reduced, thus improving the overall usability of the sensor recording software [44,50]. With Docker’s ability to define restart policies for its containers, reliability can be guaranteed in the form of prolonged runtimes [51].

**2. The *NoSQL* database *MongoDB***

- The *NoSQL* [40] database *MongoDB* [52] offers fast writing operations and is thus well suited to persisting high-frequency time-series data over a prolonged period, such as sensor recordings [40,53]. The security aspects are further enhanced by enabling MongoDB’s native encryption functionality.

**3. The reverse proxy, *Nginx*, in combination with the concurrent web server *Node.js***

- Using the combination of *Nginx* [54] and Node.js [55] in web development has been shown to be a reliable choice for the efficient handling of input/output operations (eg, handling a large number of transmission control protocol sockets due to incoming sensor requests) while ensuring performance [56,57].

**4. The column-oriented data format *Apache Parquet***

- Data in the *Apache Parquet* [58] format are stored consecutively on the hard disk and are thus highly compression friendly. This compression aspect makes Parquet particularly useful for applications that need to store and query large amounts of data (such as those generated in the analysis of time series produced in sensors) to improve their performance [50]. This aspect makes Parquet an ideal format for exported database dumps.

#### Software Architecture: Implementation

**Figure 2:**
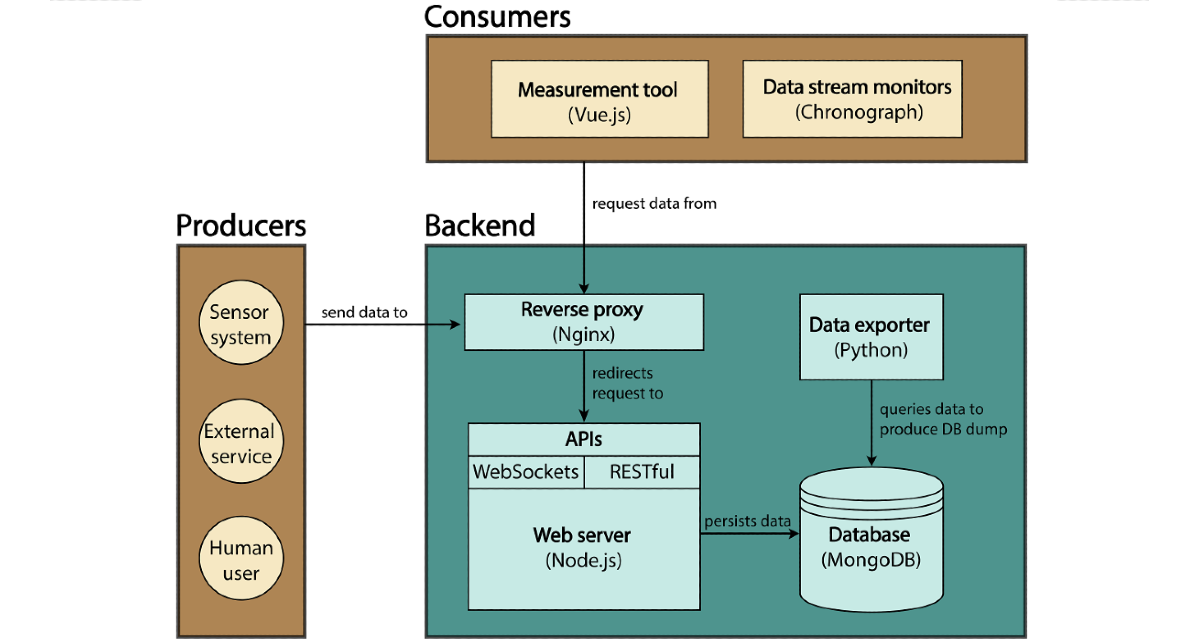
Schematic representation of the sensor recording software architecture and its functional components organized according to the client-server architecture. The backend represents the server abstraction and provides resources to its clients (consumers and producers). DB: Database.

To ensure that the SRS fulfills the previously specified nonfunctional requirements, the software is divided into modular components according to the modularity principles described by Laplante [59]. The components and their intercommunication are organized according to the client-server architecture. Consequently, the SRS architecture consists of the following three high-level components (Figure 2): the backend, as a server representing the central resource provider; the consumers, as a client fetching data streams from the server (eg, visualization programs); and the producers, as a client sending data to the server (eg, sensors, human users, or external services).

The backend is structured into functional components that each represent a collection of services that expose internal resources (eg, the database) to the application programming interface (API) end points to clients (ie, data-stream interfaces for producers and consumers). It further consists of a reverse proxy server, a web server with multiple APIs (WebSockets [60] and RESTful [55]), a database, and a data export service. Such service-oriented architecture structuring has been demonstrated to be particularly useful in the realm of web application development [56]. Moreover, all of the services are run as Docker containers and are managed via Docker Compose.

The reverse proxy, Nginx, is integrated into the backend to route client requests and to secure all communication channels with SSL/TLS (secure sockets layer/transport layer security) certificates [57]. These client requests are served by a Node.js web server. Other important tasks of this web server are the specification of client end points, the filtering of sensor streams, the setup of database connections, the scheduling of backup triggers, and the validation of the data to persist in the database. For serving client requests, the web server exposes a RESTful and a WebSocket API. The RESTful API provides end points for creating, deleting, or retrieving database entities over http, while the WebSocket API provides an interface for streaming sensor measurements to third-party applications or sending control messages to the sensor devices (eg, for toggling or rebooting) in near-real time. Furthermore, to foster their development and integration into other systems, all public-facing APIs are documented with Swagger [61]. To efficiently persist with high-frequency sensor data, the database technology MongoDB is used. Access to the end points is restricted using authentication protocols (http basic authentication and API access tokens). Data integrity constraints are guaranteed by validating the data before writing the samples into the database. The data export service is realized as a Python application that is triggered by the backend to export the acquired raw data from the database to a tabular format specified in Apache Parquet. To meet the requirements set by local ethics boards, exported files can be saved, optionally, to a long-term storage system (eg, to a network-attached storage or even a cloud storage system, such as AWS S3).

The consumer abstraction represents client applications that depend on and consume backend resources (eg, front-end applications) in the form of service applications. In this regard, several front-end applications are integrated to increase the overall usability of SRS systems. First, a graphical user interface (UI), realized as a Vue.js [62] web application, allows users to configure sensing devices, start and stop recordings, and dump the database. Vue.js was chosen because it is a single-page application framework that allows the creation of reusable components and, thus, makes it straightforward to extend the front-end application. Another consumer application included is a program used to annotate events in the time-series data assessed in a sensor recording. Next, a program to analyze the delays in persistent sensor recordings was integrated, which may help detect issues in the recording processes of sensing devices. The integration of the TICK (Telegraf, InfluxDB, Chronograf, Kapacitor) stack [63] offers further administrative simplifications that enable the SRS system to quickly store, visualize, and alert events in time-series data by integrating the following programs: Telegraph, an agent for collecting and reporting metrics; InfluxDB, a time-series database; Chronograph, a configurable dashboard to visualize InfluxDB data; and Kapacitor, an event-processing and alerting engine with bindings to notification systems (eg, via SMS text message and email). Lastly, several data processors were preimplemented to extract specific digital measures (eg, temperature statistics) from the exported data (eg, Apache Parquet files).

Regarding the security aspects, all the front-end applications are protected by means of token-based authentication techniques (eg, the OAuth2 protocol) and encrypted connections using SSL/TLS certificates, with public API requests performed over the https protocol.

The producer abstraction models data-producing client applications that interact with the backend. In other words, the producer streams data to, and receives control messages from, the backend. Producers are either sensor devices installed in the same network (eg, radars or LIDARs [light detection and ranging]), external services (eg, a producer that is in a different network, such as a commercially available cloud service), or human users who manually enter data (eg, timestamp annotations). A sensor system is either an adapter that communicates with an existing sensor device and forwards measurements to the SRS system or a driver for a sensing device (eg, radar antenna) that integrates the SRS communication protocols. In both cases, the end user must implement the adapter integration and data. Consistent communication between sensors and the backend is ensured by providing a strongly typed payload specification via JSON schema and requiring every sensor device to implement a specification to transmit data packets. This specification includes the sensor type, the structure of the sensor samples, and its expected metadata, such as the sensor’s internet protocol address. The data flow from any sensor to the backend is performed over a RESTful http API, whereas the sensor control flow, which manages the state of the sensor (such as on/off), is achieved via streaming technologies using WebSockets. Examples of sensors that have been integrated into the system using this interface are radar systems to measure gait parameters and a pressure-sensing mattress for tracking activity in bed.

The source code of the SRS is freely available on the project website [64], along with detailed documentation resources. These resources contain an outline of the architecture, a step-by-step installation manual, a Docker Compose configuration file to install and run the SRS instances, and a tutorial that describes how to integrate a new sensor.

In what follows, 3 qualitative utility programs of the SRS system are elaborated: a graphical UI to administer the SRS pipeline and mark and annotate events in a live measurement, a program to analyze delays in recorded samples, and a configurable dashboard to monitor system resources.

#### Software Architecture—Event Annotation Program

**Figure 3:**
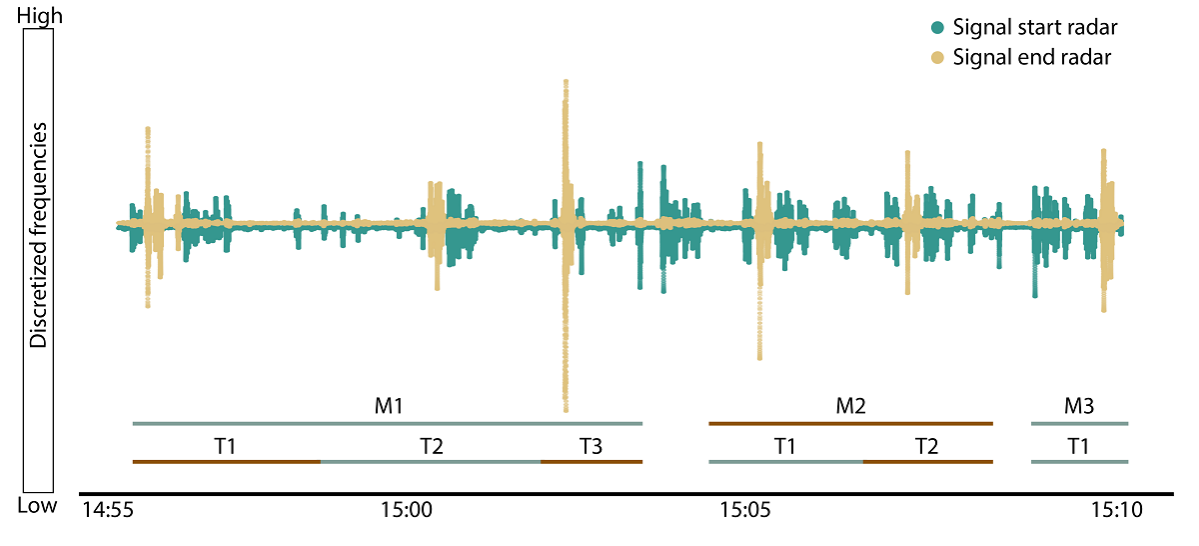
A visualization of 6 walks performed by a single participant, measured by 2 Doppler radars (indicated by the green and yellow dots), together with annotated events (ie, a correspondence between timestamp ranges, experiment, and their trial numbers).

To start and stop sensor recordings, a browser-based program with a graphical UI was integrated into every SRS system. This program allows an experimenter to manually annotate events of interest in a synchronized manner across all incoming sensor recordings. Two synchronized radar recordings were continuously assessed for 15 minutes in a measurement in November 2021 (Figure 3). The events were annotated with labels for their measurement (M1, M2, and M3) and their repetitions were expressed as trials (T1, T2, or T3).

#### Software Architecture: Sample Delay Analysis Program

**Figure 4:**
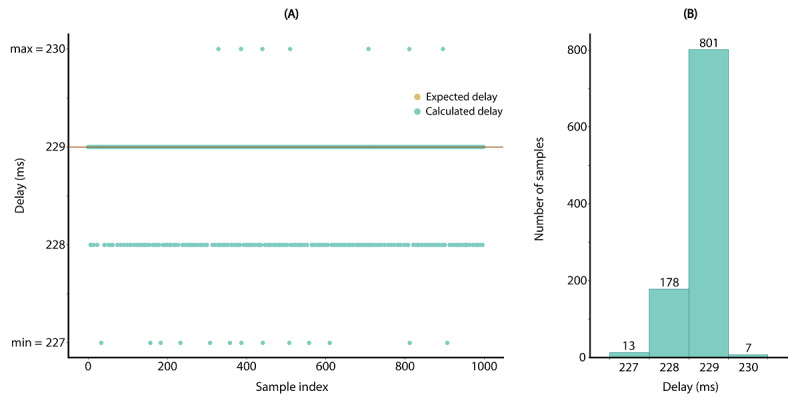
Visualization of a radar timestamp delay distribution, along with descriptive statistics of the measurement. (A) Green dots show the delay per sample and brown dots show the expected value for the delay; (B) bar chart representation of the delay distribution for 800 samples.

A program was integrated into every SRS system to analyze the delays in persistent sensor recordings. Such recordings can be used to detect indications of variations and potential delays in sensor streams. An example of a radar timestamp delay distribution produced by the SRS is shown in Figure 4.

#### Software Architecture: Monitoring Dashboard

**Figure 5:**
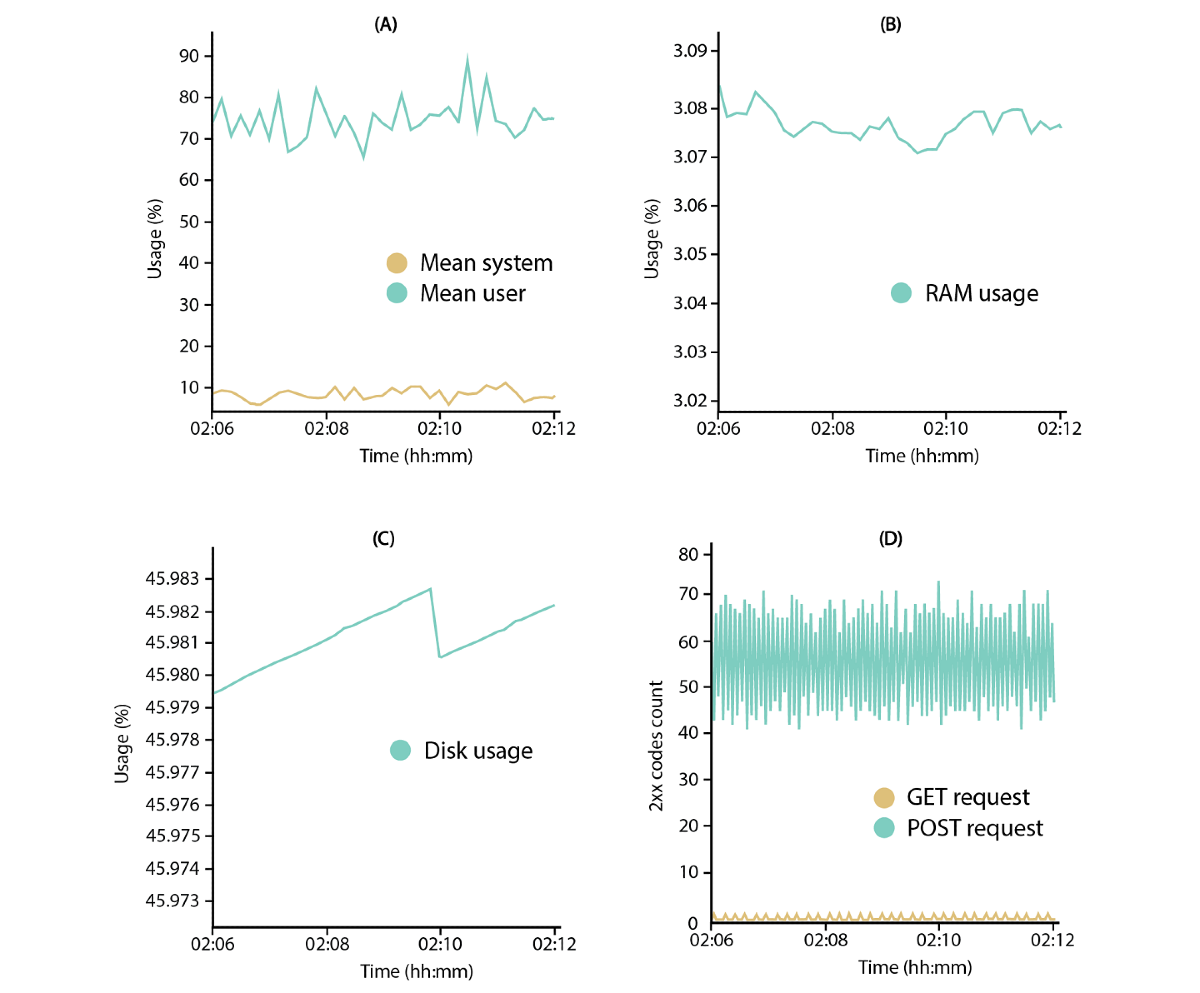
Resource monitor of the server that runs a sensor recording software instance via Chronograph dashboards: (A) CPU usage, (B) RAM usage, (C) disk usage, and (D) Nginx HTTP codes.

The TICK stack, a framework that can be used to quickly create dashboards on time-series data, was integrated to monitor various hardware and network resources of SRS systems (Figure 5). This resource monitor allows the observation of (1) central processing unit (CPU), (2) disk, and (3) RAM usage in combination with (4) the HTML status codes of network requests (ie, GET and POST).

### Field Experiments

A total of 4 SRS instances, hereafter referred to as *instance 1*, *instance 2*, *instance 3*, and *instance 4*, were used and evaluated in 3 observational studies. These studies will be broadly elaborated on to highlight how the SRS system can be used and the associated preliminary findings will be shown.

First, instance 1 was used in a study, hereafter referred to as *study 1* (n=67 for 21 days). In this study, participants had to perform various gait-related tasks in an instrumented apartment. The purpose of this study was to create a medical data set that can be used to develop novel algorithms to extract gait parameters. Second, instance 2 and instance 3 were deployed in a study, hereafter referred to as *study 2* (n=43 for 20 days), in which the system was expanded to work as a mobile and portable SRS to act as a mobile gait analyzer and to track activities of daily living (ADL). The purpose of this study was to analyze these activities in a population of healthy participants using different sensors. Notably, the results of both studies, study 1 and study 2, have yet not been published. Lastly, instance 4 was used in a pilot case study, hereafter referred to as *study 3* (n=4 for 30 days), in which, Gerber et al [65] measured ADL during a 12-hour overnight stay in an instrumented apartment. The purpose of this use case study was to demonstrate that the proposed system can be used to continuously assess digital biomarkers (eg, gait parameters and ADL) during a prolonged period and therefore represents a reliable measuring method for conducting future clinical studies in the apartment (eg, measure the change in motor functions in patients with Parkinson disease or multiple sclerosis).

Study-specific demographic information, a list of installed sensors, and the hardware specifications of each SRS instance used in each study can be found in Table 2. All sensor systems were assembled using consumer-grade hardware, without the need for any connection to the internet.

To date, a variety of different sensors have been tested and integrated into the SRS system, demonstrating the flexibility of the sensor interface. The sensors used to generate the data sets (Table 2) were Doppler radars, seismographs, LIDARs, magnetic door sensors, a motion tracking camera system, a pressure mattress for tracking activity in bed, an infrared camera, night and day cameras, passive infrared sensors, a microphone system, devices for measuring power consumption, and environmental sensors (eg, temperature, humidity, and water flow sensors). These sensors have been shown to be particularly useful in biomedical research concerning the analysis of motor and nonmotor functions [65,66] and were thus integrated into the SRS system. The performance of the SRS was statistically evaluated based on the data collected through deployed instances used in the studies. Finally, to demonstrate that the SRS has a wide area of application with respect to different hardware configurations, the software was tested on a Raspberry Pi 4 in combination with 3 radar sensors. This SRS instance was run for 24 hours with its radars configured to send 1 data packet per second from the sensors to the backend. In this proof-of-concept test, only the error rates of incoming packets and the CPU utilization were analyzed.

**Table 2:** The sensor recording software instance specifications related to the demographics, sensors, and hardware used in different studies.

Specifications			Study 1		Study 2					Study 3
			Instance 1		Instance 2		Instance 3		Instance 4	
**Participants**										
	Participants, n	67		43		43		4		
	Age (years), mean (SD)	35.0 (12.0)		34.0 (10.0)		34.0 (10.0)		32.0 (2.3)		
	Female, n	32		18		18		2		
	Experiment duration in days, n	21		20		20		30		
**Sensors**										
	Radars, n	14		N/A^a^		8		7		
	LIDARs^b^, n	5		2		2		4		
	Seismographs, n	3		N/A		2		2		
	Bed pressure sensors, n	N/A		N/A		1		1		
	Door sensors, n	N/A		N/A		N/A		23		
	Environmental sensors, n	N/A		N/A		N/A		6		
	Waterflow meters, n	N/A		N/A		N/A		4		
	Cameras, n	7		N/A		N/A		6		
	Motion tracking systems, n	1		N/A		N/A		1		
	Microphones, n	N/A		N/A		N/A		5		
	Power meters, n	N/A		N/A		N/A		13		
**Computer specifications**										
	Central processing unit (GHz; number of cores/number of threads)	3.4 (16/32)		4 (9/18)		3.9 (4/8)		4 (6/12)		
	RAM (GB)	64		32		32		32		
	Network bandwidth (Mbit/s)	1000		100		100		1000		

^a^N/A: data not applicable.

^b^LIDAR: light detection and ranging.

### System Properties

#### Overview

The quantitative performance of the SRS architecture was evaluated by statistically analyzing the data obtained in the field experiments. To compute these statistics, the system properties and associated metrics listed in the following sections were used.

#### Reliability

Following the analysis techniques outlined by Nagappan [67], the malfunctioning rate was determined by statistically analyzing the SRS log files (error, warning, and info logs) of the web server, the reverse proxy, and the database of the backend. Descriptive statistics for the error rates were determined by examining the backend logs of all instances and counting the HTML response codes. To do this, all 4xx and 5xx status codes were aggregated and counted as an error, while all remaining status codes were counted as valid requests. Based on the work of Horner and Symons [68], an acceptable error rate of 0.02% was chosen. In addition, the continuity of measurements and the potentially induced delay were calculated based on the packet timestamps of persistent samples produced by a high-frequency data producer. For this purpose, we extracted and descriptively analyzed timestamps from corresponding log files that were generated by a LIDAR sensor with a sampling rate of 40 Hz.

#### Throughput

The rate at which data were processed within a specified period was used as a performance indicator. This value was calculated by counting the number of processed SRS requests within a certain period using the program httperf [69]. This standardized tool directly outputs descriptive statistics (ie, values for the minimum, maximum, median, mean, and SD) of a performance measurement. Throughput measurements were performed for 2 http request types: GET requests (ie, fetch data from the backend) and POST requests (ie, send data to the backend). Three different tests per request type and per instance were performed. In each of these tests, http requests were sent to a randomly selected number of end points corresponding to the request type. Different numbers of requests were then sent at different request rates based on the following 2 rationales: First, every SRS instance is expected to support at least 20 concurrently running sensors sending data at 1 Hz. Second, according to performance measurements reported by Nginx, 1 core of current consumer hardware CPUs (eg, Xeon CPU E5‑2699 v3 @ 2.30 GH) is expected to be able to handle more than 400 connections per second [70]. Consequently, the following test scenarios were run and statistically evaluated: 10,000 requests at a rate of 400 requests per second (test A), 2000 requests at a rate of 100 requests per second (test B), and 400 requests at a rate of 20 requests per second (test C). Each test was repeated 20 times per instance and the average request time was determined via descriptive statistics. Notably, if a request time is below 0.1 seconds, users feel that the system is reacting instantaneously [71]. Thus, the throughput measurements were compared with this threshold and considered tolerable if their average time per request was below this value. Finally, a 2-sided *t* test (paired) was performed to compare the throughput between GET and POST requests.

#### Latency

The latency of the instances was determined by measuring how long packets took to reach their destination, also known as delay, with the program Pingmesh [72]. For this purpose, the 4 instances were repeatedly pinged, 10 times per day for 1 week. Each such measurement was performed for 120 seconds at a rate of 1 sample per second. As most of the integrated sensors communicate via Wi-Fi, the computer used to ping the instances was connected via the same connection type to the network (ie, it was not connected via Ethernet). To determine whether the SRS corresponds to a low-latency system, a delay of 100 ms was used, as this was the delay threshold for control traffic in smart grid defined in a study by Jiang et al [73]. The system latency was determined by aggregating the individual delays introduced via the systems.

#### Usability

The usability was assessed based on the responses of 10 administrators to the System Usability Scale (SUS) questionnaire [74] and the Post-Study System Usability Questionnaire (PSSUQ) [75] (Multimedia Appendices 1 and 2). The SUS is scored on a scale of 0-100, where 0 represents poor performance, 71.1 represents acceptable performance, and 100 represents excellent performance. The score of the PSSUQ is on a Likert scale that ranges from 1 to 7, with lower values indicating a better test performance. An average PSSUQ score (for its overall variable) of 2.62 or less is considered to correspond to an acceptable test performance [76]. Prior to completing the questionnaires, the administrators were asked to set up a new SRS instance, integrate a sensor, and start a sensor recording. The administrators were provided with step-by-step instructions, an SRS installation manual, a computer with a clean Ubuntu and Docker installation, and a radar sensor. During each test, the time that the participants needed to complete their tasks was recorded. In addition, after completing the test, participants were asked to provide feedback on what could be improved to enhance the overall user experience. The precise protocol for the usability test can be found in the project repository [64].

### Ethics Approval

The referred case studies were conducted according to the guidelines of the Declaration of Helsinki and approved by the Ethics Committee of the Canton of Bern, Switzerland (Kantonale Ethik Kommission [KEK] numbers 2020-02771, 2021-00965, and 2021-01420).

## Results

### Overview

This section details the statistical aspects of the analyzed system properties and experimental findings of the SRS on a Raspberry Pi 4. Next, quantitative findings in the form of the statistical outcomes of the evaluated system properties (reliability, throughput, delay, and usability) are presented. Finally, the results of a proof-of-concept 24-hour measurement performed with a Raspberry Pi 4 are demonstrated. A summary of our main quantitative findings, together with reference values from the literature, is presented in Table 3.

**Table 3:** A summary of our main findings on the analyzed system properties. For each system property, the measured average value, together with a reference value from the literature, is listed. The values of all the measured system properties were within their reference ranges.

System property	Measured value in experiments	Reference value from the literature
Reliability	Error rate of ≤0.012%	Error rate of ≤0.02% [68]
Throughout	Throughout of ≤60.69 ms	Throughput of ≤100 ms [71]
Latency	Delay of ≤12.21 ms	Delay of ≤100 ms [73]
Usability	SUS^a^ score of ≥80; PSSUQ^b^ score of ≤1.90	SUS score of ≥71.1 [74]; PSSUQ score of ≤2.62 [76]

^a^SUS: System Usability Scale.

^b^PSSUQ: Post-Study System Usability Questionnaire.

### System Properties: Reliability

HTML response codes were logged and analyzed for more than 420 days to determine the reliability performance (Table 4). In total, more than 320 million requests were sent across all instances, with an average rate of approximately 9 requests per second. A total of 7342 errors occurred, and thus, the average and maximum error percentages were low (0.002% and 0.012%, respectively). Both error percentages were within the threshold for an acceptable error rate (0.02%) [68].

During our experiments, a total of 19.8 million LIDAR packets were processed by all instances of the SRS. Their internal processing times are listed in Table 5. The mean package processing time for all instances was 25.09 (SD 0.15) ms.

**Table 4:** Descriptive statistics of HTML status codes extracted from log messages produced by our instances over 420 days. Status codes were categorized as valid (1xx, 2xx, and 3xx codes) or error (4xx and 5xx codes), and their percentage relative to the total number of received requests is reported.

Instance	Requests, n (n=323,773,157)	Valid, %	Errors, %	Runtime (days) (n=420.6)	Frequency (Hz), mean
1	268,174,824	99.998	0.002	126.1	24.6
2	23,513,498	99.999	0.001	67.4	4.0
3	26,524,834	99.988	0.012	225.1	1.3
4	5,560,001	100.000	0.000	2.0	31.9

**Table 5:** Descriptive statistics of the LIDAR (light detection and ranging) packet processing time evaluated in different instances. Each LIDAR sends small but highly frequent packages (40 Hz).

Instance	Packets, n (n=19,817,460)	Time (ms), mean (SD)
1	8,227,508	25.08 (0.15)
2	1,603,094	25.09 (0.15)
3	3,830,283	25.07 (0.17)
4	6,156,575	25.10 (0.12)

### System Properties: Throughput

**Figure 6:**
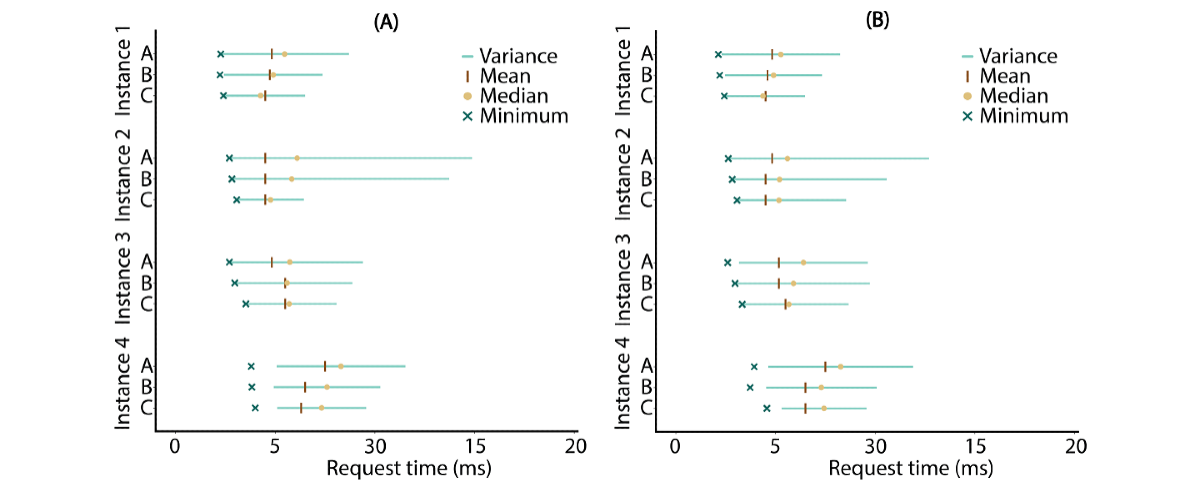
Descriptive statistics of (A) GET and (B) POST requests. The y-axis represents the different combinations of reference instances (Instance 1 to Instance 4) and test scenarios (test A to test C). The x-axis indicates the time it took an instance to process the received request (depending on the test scenario). Horizontal lines indicate the variance, while vertical bars indicate the mean. The median is represented by a dot, and a cross indicates the minimum. Maximum values were omitted from the figure to improve the overall clarity, as these values were outside the plotted scale.

Test results of GET and POST throughput experiments are shown in Figure 6. For both request types, across all experiments, the average request time was short, ranging between 4.72 and 8.30 ms (mean 5.98 ms, SD 1.82 ms). Furthermore, independent of the instance and request type, on average, as the number of requests increased, the SD of the throughput rate increased (test A: SD 48.00 ms; test B: SD 3.84 ms; test C: SD 2.39 ms). Similarly, the average maximum throughput rate increased (test A: maximum 60.49 ms; test B: maximum 54.00 ms; test C: maximum 25.90 ms). Both request types reported very similar and low average minima (GET: minimum 3.28 ms; POST: minimum 3.29 ms). The average and maximum request times (5.98 and 60.49 ms, respectively) were below the threshold of an instantaneously reacting system (100 ms) [71]. For all instances, the median was very close to the corresponding mean, with an average difference of 0.67 ms. There was no significant difference (*t*_480_=–0.0571; *P*=.045) in throughput between GET (mean 5.97 ms, SD 3.64 ms) and POST (mean 5.99 ms, SD 3.50 ms). No packet losses were observed during the testing.

### System Properties: Latency

**Figure 7:**
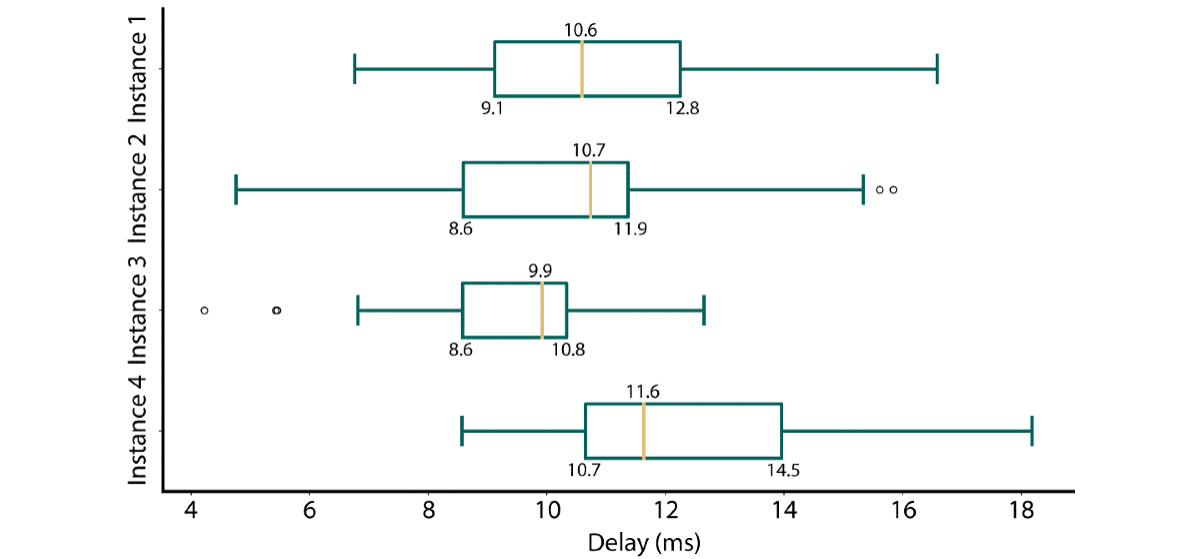
Box plot visualization of the per-instance delay values.

The evaluation of instance latency was realized via box plot representations of long-term delay measurements (Figure 7). Across all instances, the average delay was low (between 9.9 and 11.6 ms, mean 10.7 ms, SD 2.0 ms). Instance 3 exhibited the lowest delay (mean 9.55, SD 1.33 ms), and instance 4 exhibited the highest delay (mean 12.21 ms, SD 2.18 ms). The average delay across all instances (10.88 ms) was below the definition of a low-latency system (100 ms) [73]. Moreover, there were no significant differences in delay between the instances (*t*_33,600_=–1.54 × 10^–15^; *P*<.001). Finally, few outliers were reported, and no packet losses were observed during the testing.

### System Properties: Usability

**Figure 8:**
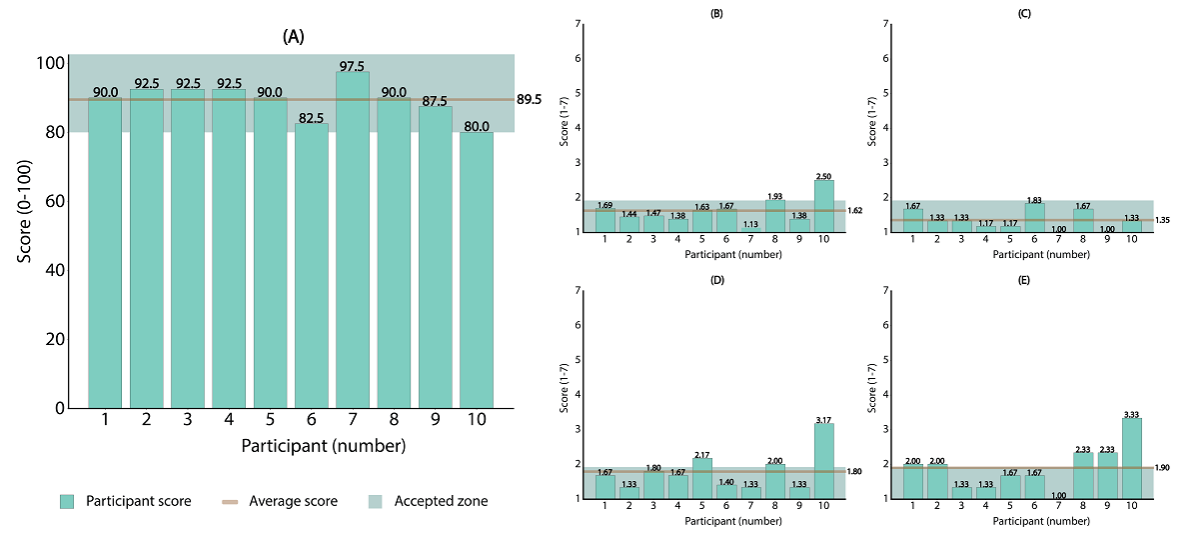
(A) The SUS score for each participant, with the brown line indicating the average SUS score across all participants (mean 89.5, SD 4.8). A score of 100 represents excellent usability, while lower scores represent poor usability. Panels B to E represent the PSSUQ scores (ie, Overall, SU, InfQ, and IntQ) for each participant, with the brown line indicating the average value. The PSSUQ scores range between 1 (best value) and 7 (worst value). The green colored areas in the subfigures correspond to scores that are within the specified threshold values (ie, the accepted zone). InfQ: information quality; IntQ: interface quality; PSSUQ: Post-Study System Usability Questionnaire; SU: system usability; SUS: System Usability Scale.

The system usability was assessed by analyzing the responses of 10 participants to the SUS and PSSUQ (Figure 8). The mean participant SUS score yielded an excellent (>80.3) usability rating [74], with scores between 80.0 and 97.5 (mean 89.5, SD 4.8). The mean overall PSSUQ score (mean 1.62, SD 0.36), as well as its system usability (mean 1.35, SD 0.27), information quality (mean 1.80, SD 0.54), and interface quality (mean 1.90, SD 0.63) scores attained an acceptable level (overall: <2.62) [75,76].

According to the participants’ qualitative feedback, the installation script was helpful and simple to use. By contrast, the experiment UI could be made easier to use by adding colors to functional components, such as buttons and drop-down menus.

### Field Experiments: SRS on a Raspberry Pi 4

The SRS was installed on a Raspberry Pi 4 in combination with 3 radar sensors. The resulting system was run for 24 hours. In the measurement, only reliability aspects, along with CPU usages, were analyzed. The system did not report any errors during the measurement. In total, more than 250,000 packets were processed under high load (CPU usage: mean 95%). No packets were lost during the measurement.

## Discussion

### Principal Findings

In this paper, we reported the development, functional components, and performance evaluation of a novel lightweight, open-source, and cost-efficient software system. This system was built to enable sensor data collection that offers both cloud and on-premises deployment. In line with our objective, the SRS can be reliably used to integrate multiple sensors on consumer-grade hardware to perform offline measurements in clinical studies. This was shown by analyzing different performance metrics applied to various long-term measurements.

To demonstrate that the SRS helps to address technical challenges that arise during multimodal sensor measurements (eg, unsynchronized sensor signals), the following three utility programs were developed and integrated: (1) a timestamp-annotation program to mark events that are synchronized across measurements; (2) resource monitoring software, called the TICK stack, to alert administrators to the presence of hardware errors related to the server, nonfunctioning sensors, or network problems; and (3) a timestamp analyzer to detect and understand delays in the recorded data due to potential sensing device issues.

With respect to the quantitative findings, it was found that the SRS system operated with an acceptable error rate while running at a high request rate. This was determined by analyzing the error rates of more than 320 million requests transmitted over 420 days and relying on Horner’s definition of acceptable error rates [68]. It is noteworthy that most errors arose due to problems in the power supply to the system caused by a power outage that lasted several hours. Moreover, a long-term measurement of the internal packet processing time showed a low deviation in time. This indicates that the SRS system was internally processing data packets at a stable rate. Those two findings are in line with our objective, which was to make the internal system operate reliably.

Next, the throughput performance of the system was examined. The results demonstrate that, regardless of the request type, the SRS was capable of efficiently functioning at a high request rate (2000 requests per second) on consumer-grade hardware. Based on the literature, the results meet the criteria of an instantly responding system [71]. This means that the SRS system can handle high-frequency data streams in real-time and on consumer-grade hardware, which makes the system promising for monitoring. Furthermore, for all the systems and request types, the average request time was determined to be close to the median request time. This is an indicator of stable request processing, suggesting that the throughput rate was stable over a prolonged period. This processing property leads to the conclusion that the throughput rate of serving incoming API requests was stable over a prolonged period.

In addition to running stably, the delay results indicate that the SRS corresponds to a low-latency system, as defined by Jiang et al [73], which implies that the network topology does not affect the transmission rates between the sensors and the SRS. Furthermore, the average SD was small, which indicates that the latency was stable over time. Both findings imply that the SRS did not introduce any significant delay during the sensor recordings and, thus, did not affect the measurement process.

The overall usability was assessed, including through the rating of the complexity of setting up an SRS instance and its applicability for measurements. An SUS score indicating excellent usability was achieved [74], suggesting that the system is highly usable. Moreover, according to the results of the PSSUQ, the usefulness, information quality, and interface quality of the SRS system are at acceptable levels [75], indicating that the UI is easy to use and exhibits helpful information pertaining to its use. Furthermore, the results of the 2 usability tests show that, in line with the initial objective, the overall usability of the SRS is effective, efficient, and satisfactory. The participants particularly appreciated that the SRS can be installed via Docker, as it simplified the installation effort. However, a major weakness identified by the participants was the unintuitive nature of the UI components. If this issue is eliminated, the overall usability of the interface could be improved.

Finally, to demonstrate the SRS’s broad integration capability, it was operated on a Raspberry Pi 4, in combination with several integrated sensor devices, over a period of 24 hours. During this period, no packets were lost, and no errors were reported in the logs. However, due to the exploratory nature of this test, no other system properties were analyzed. Nevertheless, this finding indicates that the SRS could be a promising software solution with which to conduct biomedical research on cost-efficient consumer-grade hardware as basic as a Raspberry Pi 4.

### Strengths and Limitations

The SRS system offers a simple and cost-efficient solution for on-premises deployment that requires only minimal technical expertise and provides high usability. The SRS may provide numerous benefits for researchers conducting early stage research, or working with limited resources, compared with cloud-based solutions [23,24,27] that require on-premises deployment and normally face economic, technical, and regulatory challenges. This is mainly because the SRS runs stably on a variety of consumer-grade hardware, even as basic as that on a Raspberry Pi 4. It is also due to the fact that the system supports a simple mechanism for integrating multiple and diverse high-frequency data streams from various sensing devices. The proposed system is therefore a feasible option for long-term measurements that takes into account biomedical research requirements, such as privacy protection. Integrating Docker in the system may help users of the SRS replicate studies as the utilized containers consist of replicable configurations (ie, the source code of the executed program, the execution order in form of a Dockerfile, and defined environment variables). Furthermore, by providing a digest of the data stored in the database of a particular study (eg, by exporting a database dump or hashing the persisted data), users of the SRS are not only able to replicate that study, but also able to reproduce it. Additional aspects of the system enable straightforward deployment and enhanced maintainability while easing its operationalization (ie, integration and management). It further promotes proper error handling, which reduces the complexity of the run-time diagnostics used to determine the cause and extent of errors. Consequently, this lightweight recording software system allows researchers to focus resources on research questions rather than on developing technology.

Although it was successful in reaching its desired objective, the SRS system still has some limitations. For example, although a variety of different hardware combinations were tested, the extent to which these results can be extrapolated to low-cost hardware (eg, ESP32 [Espressif Systems 32 Microcontroller] microcontrollers) is not clear. From a performance perspective, this becomes particularly challenging when integrating many sensors, as the hardware specifications of low-cost systems may be too low to handle a large number of incoming requests. Furthermore, although the SRS achieved high scores for usability, the generalizability of these results is limited by the low number of participants. Another usability hurdle is the integration of new sensor types, as this currently requires adherence to interface specifications, which can be tedious.

### Future Work

To expand the system for applications beyond clinical needs and to mitigate potential scaling issues, the SRS could be extended to scale horizontally by operating as a distributed service in the cloud. Future studies could investigate how, and to what extent, the SRS system could be extended to properly scale in multiple clinical settings, in which many SRS instances might be running simultaneously while sharing a central database. In addition, the performance could be improved using streaming technologies (eg, broker-based solutions). To benchmark the system, the methods used to measure the performance could be further improved by correlating the measurements with more system resources (eg, by comparing the throughput with the CPU and RAM usage). Finally, the sensor integration could be simplified by relying on standardized serialization mechanisms to represent structured data (eg, Google’s Protocol Buffers). This would reduce the effort to integrate new sensor devices even further.

### Conclusion

Despite the significant potential of these new digital technologies, adopting sensor-enhanced biomedical solutions is not simple due to the complex and expensive nature of most existing SRS. This paper presents a lightweight software recording system for integrating arbitrary sensing devices on consumer-grade hardware to perform reliable long-term measurements. The system has significant potential to address the economic, technical, and data regulatory challenges associated with earlier systems, thereby enabling sensor measurements and objective assessments in the realm of biomedical research. Moreover, the system facilitates the testing of sensor systems, as well as the development and validation of algorithms for the extraction of digital measures. Overall, this allows researchers to focus on their research questions rather than on developing the technology needed to collect their data.

## Appendix

Multimedia Appendix 1 SRS usability test. SRS: sensor recording software.

Multimedia Appendix 2 SRS usability test protocol. SRS: sensor recording software.

## Abbreviations

ADL: activities of daily living
API: application programming interface
CPU: central processing unit
ESP32: Espressif Systems 32 Microcontroller
LIDAR: light detection and ranging
mHealth: mobile health
PSSUQ: Post-Study System Usability Questionnaire
SRS: sensor recording software
SSL: secure sockets layer
SUS: System Usability Scale
TICK: Telegraf, InfluxDB, Chronograf, Kapacitor
TLS: transport layer security
UI: user interface

## Data Availability

The data sets generated during and analyzed during this study are not publicly available due to privacy and local ethical restrictions but are available upon request.
