# Supplementary material for: Development of an Open-source and Lightweight Sensor Recording Software System for Conducting Biomedical Research: Technical Report
Source: JMIR Form Res. 2023 Feb 17;7:e43092. doi: 10.2196/43092 (PMC9985000; doi:10.2196/43092)

SRS Usability Test

In this usability test, you will be asked to install the Sensor Recording Software (SRS) on a provided computer, integrate a radar sensor, set up an experiment and start a recording for that experiment. To do so, you must follow the instructions described in the enumerated steps in order. After completing the test, you will have to fill out two questionnaires with questions regarding the system's usability.

Please make sure that you read the instructions carefully, follow the instructions in order, and do not skip any step. Make sure that you had a close look at every provided screenshot to properly understand the relevant steps. This document consists of nine pages in total.

You are allowed to ask the operator any question regarding the provided computer system (e.g., how to open a browser or how to open terminal) but no question that help regarding the step regarding solving one of the tasks in this test.

Part 1: Installation

1. Log-in into the server via SSH:
    a. Open the terminal on your computer and enter:
        i. **ssh smdp01@192.168.30.100**
        ii. Use the following password: **artorg1234**
2. Clone the SRS project via git:
    a. **git clone** git@gitlab.com:sensor-monitoring/srs.git
3. Change directory: **cd srs**
4. Fetch all submodules: **git submodule update --init --recursive**
5. Install the SRS software: **sudo bash quick_install.sh**
    a. Enter the password **artorg1234**

Part 2: Extend Sensor Configuration

Enter a new radar sensor to the system by performing the following steps:

1. Execute **sudo bash add_sensor.sh**
2. Follow the dialogue:
    a. Select the radar type by entering **11**
    b. Enter **192.168.30.102** as IP address
    c. Enter **office** as location
    d. Enter **radar01** as name
    e. Press **Enter** to exit the program
3. Restart the pipeline by executing **sudo bash quick_restart.sh** in your terminal.

Part 3: Login and Create a New User

1. Open a browser of your choice and enter the following URL: **http://192.168.30.100:3000**
2. Use the login credentials **admin** with password **123456**
3. **Create a new User (See screenshots below):**
    a. Click on the *Users* Menu entry
    b. Click on *CREATE NEW USER*
    c. Fill-in the data shown in the screenshot below, click on the drop-down for selecting *Roles*, and the *Password* **123456**

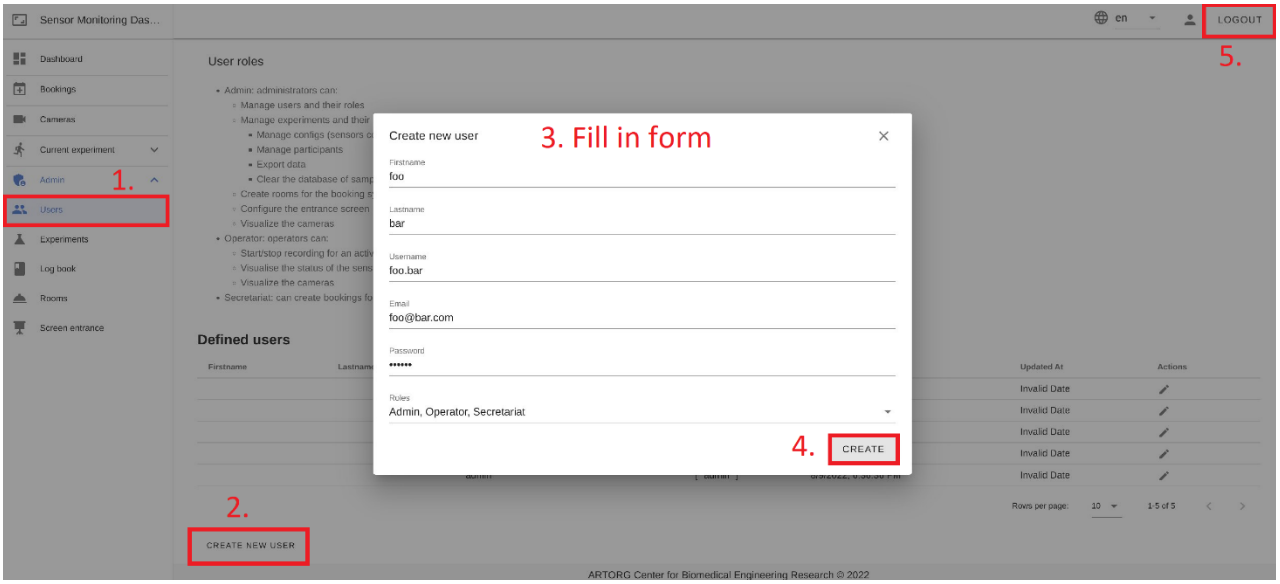

4. Click on the button *CREATE*.
5. Click on the *LOGOUT* button on the top right.
6. Enter the credentials (user: **foo.bar**, password: **123456**) of the user you just have created to log-in again.

Part 4: Create an Experiment

Create a new sensor Configuration (See screenshots below)

1. Click on the _Admin_ Menu entry (in menu bar on the left-hand side), click on the sub-menu entry _Experiments_.
2. Select the Tab *CONFIGS*
3. Click on the button *CREATE NEW CONFFIG*
4. A pop-up appears: Enter **test** in the text-field *New config name*.
5. Click on the button *CREATE CONFIG*.

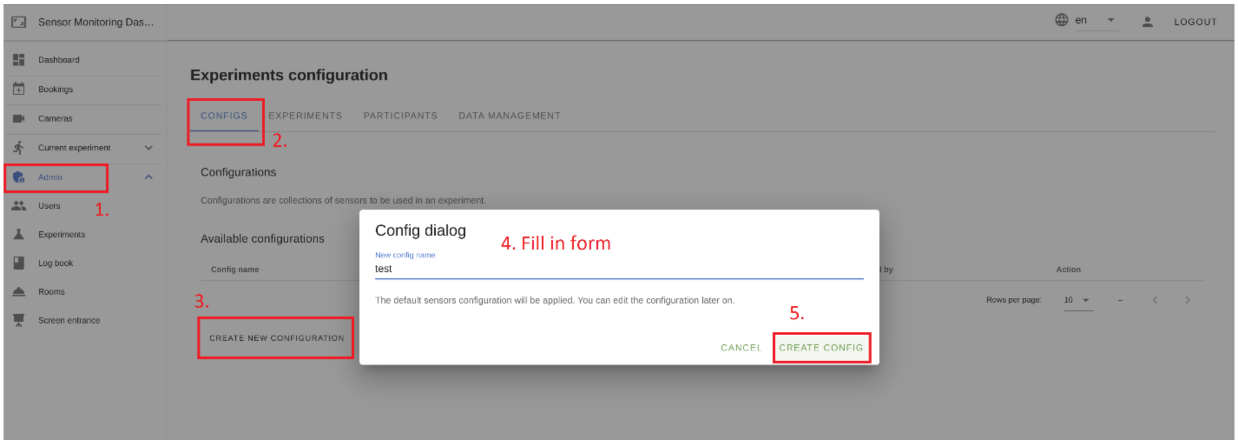

6. Next, click on the button *LOAD* next the just created configuration *test*.
7. Scroll down until you reach the *radar_sensors* table.
8. Select the toggle *Save data* as shown in the screenshot below.

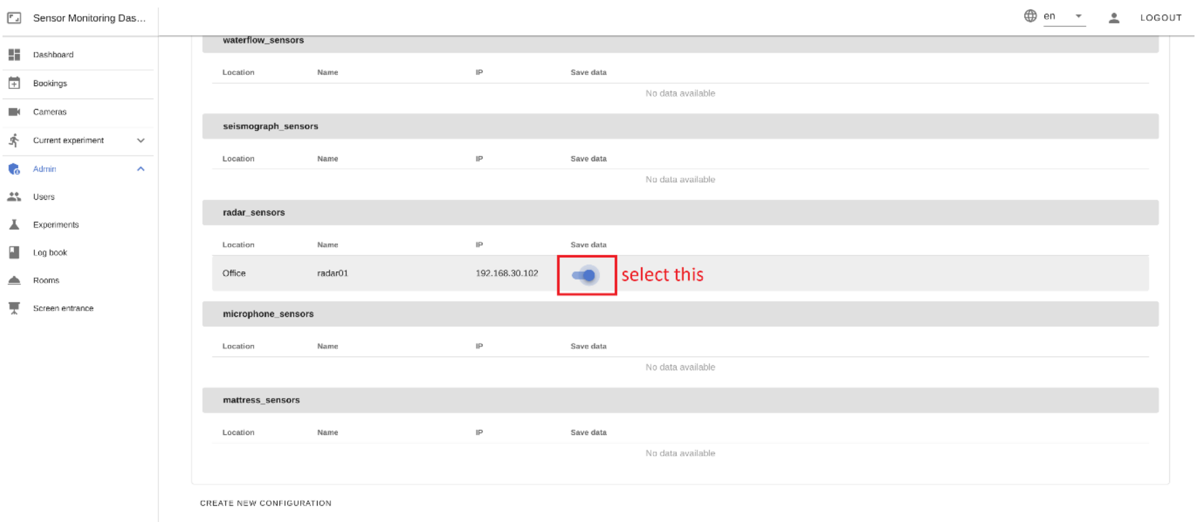

9. Scroll up and click on the blue button *UPDATE.*

Create a new Participant (See screenshots below)

1. Click on the *Admin / Experiments* Menu entry (if not already selected)
2. Select the Tab *PARTICIPANTS*
3. Click on the button *CREATE NEW PARTICIPANT*
4. A pop-up appears: click on the wizard wand on the right to create a UUID and use the short name **p01**,
5. Click on the button *CREATE*.

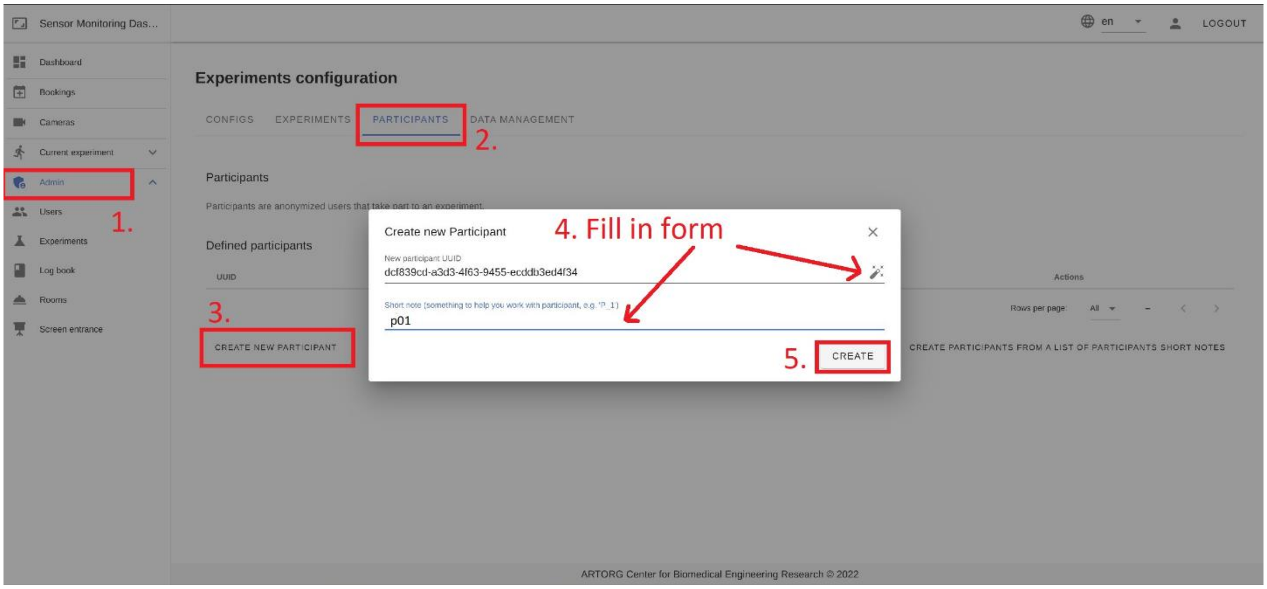

Create a new active Experiment

1. Click on the *Admin / Experiments* Menu entry (in menu bar on the left-hand side)
2. Click on the button *CREATE NEW EXPERIMENT*
3. A pop-up appears (see screenshot below):
   a. Enter **test** in the text-field *New experiment name*.
   b. Click on the wizard wand on the right to create a UUID
   c. Select the *Configuration* **test** by clicking on the dropdown on the right.
   d. Select **foo bar** as *Owner* by clicking on the dropdown on the right.
   e. Click on the button *CREATE EXPERIMENT*.

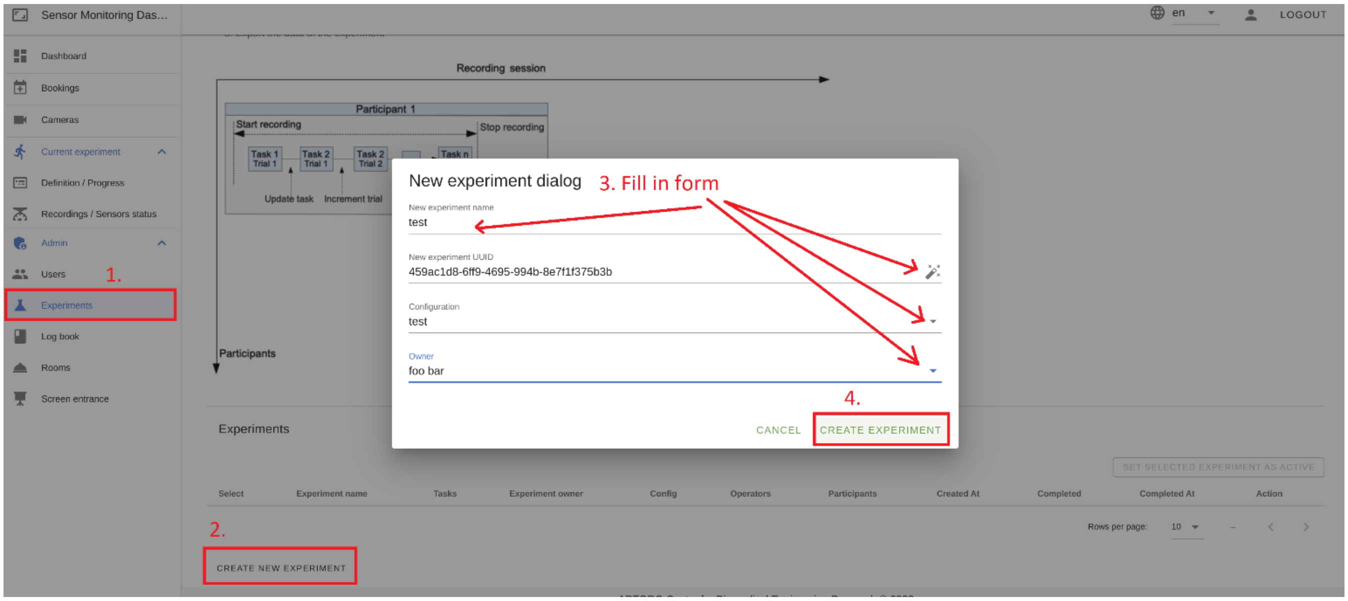

4. Click on the *Select* radio button (on the left-hand side of the newly created experiment **test**)
5. Click on the button *SET SELECTED EXPERIMENT AS ACTIVE*
6. Click on the button *EDIT*, a pop-up appears.

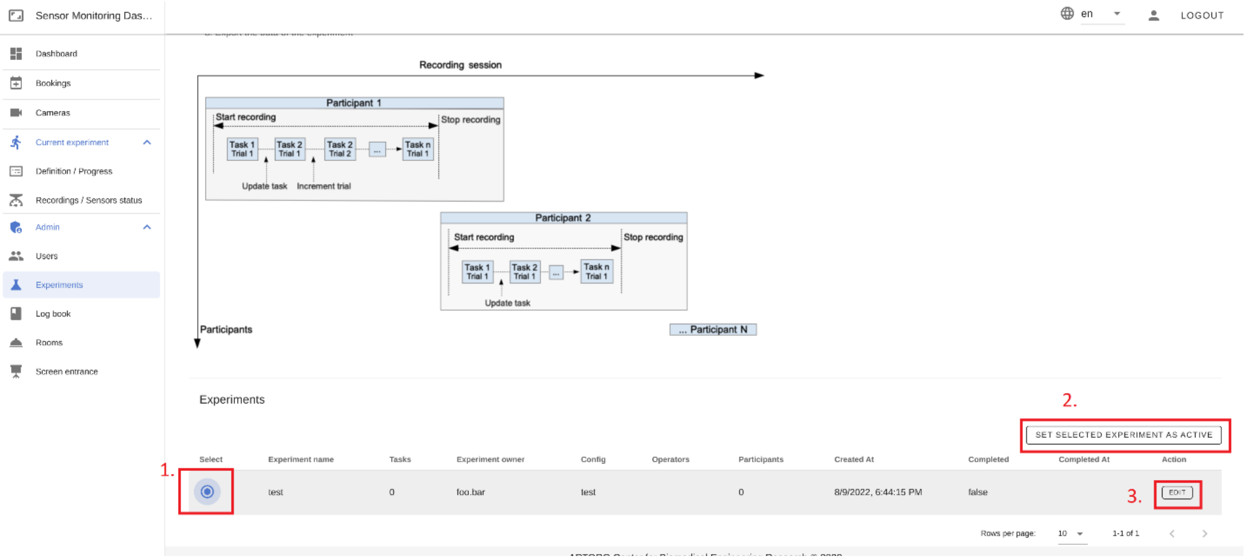

7. Scroll down until you reach the categories ***Task***, ***Operators*** and ***Participants***.

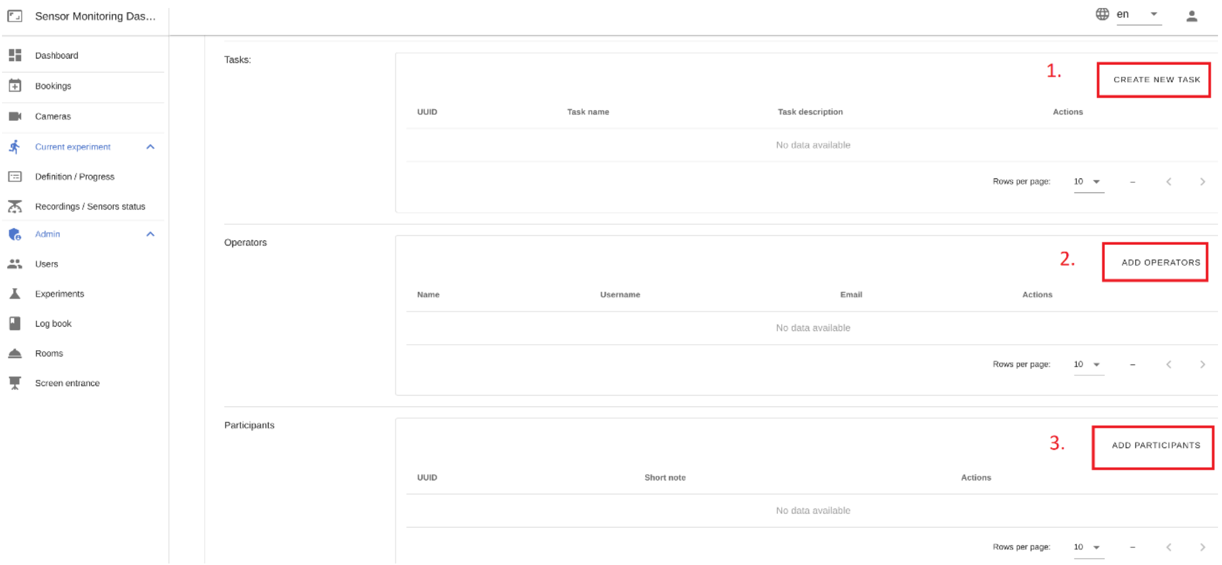

8. Click on the button *CREATE NEW* Task, a pop-up window appears:
   a. Click on the wizard wand on the right to create a UUID
   b. Enter **Run01** in the text-field Task name
   c. Click on the button *CREATE*.
9. Click on the button *ADD OPERATORS*, a pop-up appears:
   a. Use the selector *Select operators to add* and check the option **foo.bar**
   b. Click on the button *ADD*
10. Click on the button *ADD PARTICIPANT*, a pop-up appears:
    a. Use the selector *Select participants to add* and check the option **p01**
    b. Click on the button *ADD PARTICIPANT*

Part 5: Start an Experiment Recording

1. Click on the *Current experiment* Menu entry (in menu bar on the left-hand side)
2. Next, Click on the sub-menu entry *Recordings / Sensors status*.
3. Select participant **p01** by clicking on the dropdown menu *Please select a participant*.
4. Select task **Run01** by clicking on the dropdown menu *Current Task*.
5. Click on the button *START RECORDING*
6. Wait for 30 seconds and then click on the button *STOP RECORDING*.

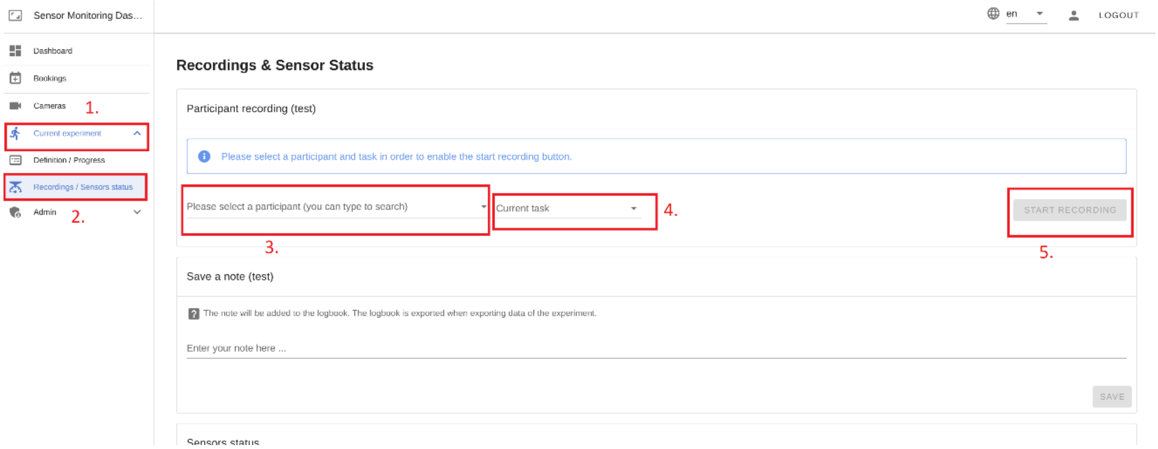

Supplement: Multimedia Appendix 1 [file formative_v7i1e43092_app1.pdf]
