# Supplementary material for: Development of an Open-source and Lightweight Sensor Recording Software System for Conducting Biomedical Research: Technical Report
Source: JMIR Form Res. 2023 Feb 17;7:e43092. doi: 10.2196/43092 (PMC9985000; doi:10.2196/43092)
Supplement: Multimedia Appendix 2 [file formative_v7i1e43092_app2.pdf]

UNIVERSITÄT BERN

SRS Usability Test Protocol

Test the Sensor Recording Software (SRS) system in a usability study. To achieve this, participants in this study must perform certain tasks.

Objective

The objective of this study is to assess the usability of the SRS system in terms of installing and using the system as a user with technical experience. We hope to find evidence of an overall pleasant user-experience and to leverage the process of conducting biomedical measurements. Furthermore, we also hope to receive feedback on potential improvements in the use of SRS.

Participants

To conduct this usability study, we want to recruit 10 participants that have prior knowledge in DevOps (combination of software developers [dev] and operations [ops]) related work (e.g., feeling comfortable using git and a Linux terminal). The recruitment will be executed via E-Mail and send to researchers and computer scientists working in Swiss research facilities.

Equipment and Material

A notebook with an Ubuntu 22.04 installation and with an internet connection will be provided. Relevant programs to perform the usability task will be installed on that machine. After performing the usability test, the participants will be asked to fill-out questionnaires (the *SUS* and *PSSUQ* questionnaires). The questionnaires are assessed and managed digitally by using the program *REDCap*.

Location and Dates

Participants are asked to come to our lab offices and perform the test on a day a scheduled date.

Test Procedure Execution

Before starting the test, participants are introduced to the provided computer system, the relevant tools (e.g., how to open a terminal) and how to open a browser (i.e., Google Chrome). Participants are then asked to carefully read the task descriptions in the provided test instruction document. Furthermore, participants are informed about the fact that they are supposed to fill out two questionnaires (i.e., the SUS and PSSUQ questionnaires) after completion of their test. Participants are allowed to ask questions regarding the provided computer system but not to obtain additional information about the SRS system to complete the task. Finally, participants are asked to sign the informed consent, before they are allowed to start the test by opening the provided test document.

After completing the test, each participant will be asked to comment on what could be improved in the SRS. During the experiment, the time it took a participant to complete the test is measured (without letting them know). Then, they are asked to fill out the questionnaires. During the experiment, the time it took a participant to complete the tasks is measured (without letting them know).

Tasks

A detailed list of all tasks all participants will have to perform can be found in a separate document (see srs_usability_test.pdf).
